# Supplementary material for: The Systems Biology Research Tool: evolvable open-source software
Source: BMC Syst Biol. 2008 Jun 29;2:55. doi: 10.1186/1752-0509-2-55 (PMC2446383; doi:10.1186/1752-0509-2-55)
Supplement: Additional file 1 — SBRT Archive. An archive of the current version of the Systems Biology Research Tool. [file 1752-0509-2-55-S1.zip › sbrt-1.4.0/doc/developers_guide/api/sbrt/kernel/math/graph_theory/class-use/DirectedGraph.html]

Uses of Class sbrt.kernel.math.graph\_theory.DirectedGraph


|  |  |  |
| --- | --- | --- |
| **Overview** \| **Package** \| **Class** \| **Use** \| **Tree** \| **Deprecated** \| **Index** \| **Help** | |  |
| PREV   NEXT | **FRAMES**    **NO FRAMES**     **All Classes** |  |


---


## **Uses of Class sbrt.kernel.math.graph\_theory.DirectedGraph**

| Packages that use DirectedGraph | |
| --- | --- |
| **sbrt.kernel.math.graph\_theory.comp** | Provides classes and interfaces for performing graph theoretical computations. |
| **sbrt.kernel.math.graph\_theory.proc** | Provides classes and interfaces for performing graph theoretical processes. |
| **sbrt.shell.mng.graph\_theory** | Provides classes and for creating graph theoretical process managers. |

Uses of DirectedGraph in sbrt.kernel.math.graph\_theory.comp

| Methods in sbrt.kernel.math.graph\_theory.comp that return DirectedGraph | |
| --- | --- |
| `DirectedGraph<N>` | `PathFinder.getGraph()`             Returns the graph in which pathways will be computed. |

Constructors in sbrt.kernel.math.graph\_theory.comp with parameters of type DirectedGraph

`PathFinder(DirectedGraph<N> graph)`
  
          Constructs a new path finder for the provided graph.

Uses of DirectedGraph in sbrt.kernel.math.graph\_theory.proc

| Methods in sbrt.kernel.math.graph\_theory.proc with parameters of type DirectedGraph | |
| --- | --- |
| `void` | `CycleIdProcess.setGraph(DirectedGraph<N> graph)`             Sets the graph for which all cycles will be identified. |
| `void` | `PathIdProcess.setGraph(DirectedGraph<N> graph)`             Sets the graph for which all paths will be identified. |

Uses of DirectedGraph in sbrt.shell.mng.graph\_theory

| Methods in sbrt.shell.mng.graph\_theory that return DirectedGraph | |
| --- | --- |
| `static DirectedGraph<java.lang.String>` | `PathIdManager.getGraph(ManagerInput<java.lang.String,java.lang.String> input, java.io.PrintWriter out)`             Opens the file denoted by `PathIdManager.EDGE_FILE` in the provided manager input and returns the directed graph it contains. |
| `static DirectedGraph<java.lang.String>` | `PathIdManager.getGraph(java.lang.String fileName)`             Parses the file with the provided name, and returns the directed graph it contains. |
| `static <N> DirectedGraph<N>` | `PathIdManager.getGraph(java.lang.String fileName, DirEdgeFormat<N> format)`             Parses the file with the provided name, and returns the directed graph it contains. |

---


|  |  |  |
| --- | --- | --- |
| **Overview** \| **Package** \| **Class** \| **Use** \| **Tree** \| **Deprecated** \| **Index** \| **Help** | |  |
| PREV   NEXT | **FRAMES**    **NO FRAMES**     **All Classes** |  |


---
